# Would Your Clothes Look Good on Me? Towards Transferring Clothing Styles with Adaptive Instance Normalization

**DOI:** 10.3390/s22135002

**Published:** 2022-07-02

**Authors:** Tomaso Fontanini, Claudio Ferrari

**Affiliations:** Department of Engineering and Architecture, University of Parma, 43124 Parma, Italy; claudio.ferrari2@unipr.it

**Keywords:** deep learning, style transfer

## Abstract

Several applications of deep learning, such as image classification and retrieval, recommendation systems, and especially image synthesis, are of great interest to the fashion industry. Recently, image generation of clothes gained lot of popularity as it is a very challenging task that is far from being solved. Additionally, it would open lots of possibilities for designers and stylists enhancing their creativity. For this reason, in this paper we propose to tackle the problem of style transfer between two different people wearing different clothes. We draw inspiration from the recent StarGANv2 architecture that reached impressive results in transferring a target domain to a source image and we adapted it to work with fashion images and to transfer clothes styles. In more detail, we modified the architecture to work without the need of a clear separation between multiple domains, added a perceptual loss between the target and the source clothes, and edited the style encoder to better represent the style information of target clothes. We performed both qualitative and quantitative experiments with the recent DeepFashion2 dataset and proved the efficacy and novelty of our method.

## 1. Introduction

Recently, deep learning has drawn lots of attention from the fashion industry due to its possible application to many different tasks. These include the automatic classification of clothes [1], which can be employed, for instance, for the automatic sorting of an online shop. Next, retrieval techniques can be used in fashion [2] to help the client find a specific item given a single picture. In addition, a good recommendation system is fundamental for basically every online shop, especially fashion ones [3], where keeping track of the user’s preferences and presenting suggestions to the client based on these can help improve sales by a great margin.

Even if all these tasks present several unresolved challenges, one that is still in an early stage regards synthesizing clothes images using deep learning. Particularly, this is due to the impressive amount of variability that characterizes the clothes image category. Clothes exist in significantly large variety of styles, shapes, textures, materials, and colors; this represents a great obstacle, even for state-of-the-art generative techniques, as in [4]. Indeed, it can be claimed that is much easier for a network to generate a realistic face than realistic clothes. Nevertheless, being able to generate realistic clothes can be of great interest for designers and stylists because it can greatly help their work and boost their creativity.

Regardless of the great challenge that this particular task brings with it, several works were presented to tackle this problem from different angles. Some works had the objective of generating a full person wearing particular clothes, as in [5]. This process was sometimes facilitated by starting the generation from a segmentation map [6]. In this case, the task is more similar to the standard semantic image synthesis with generative adversarial networks (GANs). However, it could have less applicability in the fashion industry due to the lack of control on what the network will generate.

In addition to that, recently, virtual try-on systems have drawn lot of attention [7,8]. They have the objective of fitting particular clothes on a target image of a person. They can be very useful for simulating wearing particular clothes before buying and, for this reason, are especially relevant for online shops where users do not have the chance to try a particular clothes. More specifically, virtual try-on systems usually rely on fitting specific source clothes to a target mask and completely replacing them.

In this work, we address the following question: given two images of people, one target and one source, wearing some clothes, is it possible to transfer the style (color, texture) of the target’s clothes to the source image while maintaining the original shape, e.g., dress, pants, shirt? Differently from existing works (such as [9]) which extract styles from random images such as paintings or textures and try to apply it to a single clothes image (with no corresponding person wearing it), we opt for a more challenging setting in which we extract the “clothing style” from a target *person* and transfer it to a source *person*. This is of great interest for both customer and industry, as the first has the possibility to explore different styles of clothes matching his or her taste and the latter can generate different clothes prototypes without the need of making them manually. To deal with the fact both source and target images contain people, we use a segmentation mask to separate the clothes area with the rest of the image that does not have to be considered during the style transfer.

This is performed by employing a style encoder that extracts style codes from the clothes area, and then injects these codes into a generator network consisting of an encoder–decoder structure. In more detail, the style codes are used to produce parameters for the Adaptive Instance Normalization (AdaIN) [10] layers that are present in the decoder network. Indeed, AdaIN layers allows us to transfer the target style to the source image without additional training steps during inference time. Our network architecture takes inspiration from StarGANv2 [11], though with some fundamental changes; in particular, we remove the domain separations in the model as it is not important for our style transfer task. Then, we modified the StarGANv2 style encoder architecture augmenting the style dimension and replacing two downsampling layers with a pooling function in order to remove the shape information from the style codes.

To summarize, the main contributions of the proposed work are the following:

- A system that is able to extract a “clothing style” from a person wearing a set of clothes and transfer it to a source person without additional training steps required during inference;
- The model is trained in an end-to-end matter and employs clothes masks in order to localize the style transfer only to specific areas of the images, leaving the background and person’s identity untouched;
- Extensive experiments performed on a particularly-challenging dataset, i.e., DeepFashion2 [12].

The paper will be organized as follows: firstly, in Section 2, current state-of-the art literature will be presented; then, in Section 3, the proposed architecture will be described, together with the training losses; Section 4 reports experimental results, both qualitatively and quantitatively; finally, in Section 5 conclusions and future works will be discussed.

## 2. Related Work

**Generative Adversarial Networks.** In recent years, Generative Adversarial Networks (GANS) have become the state-of-the-art for generative tasks. They were first introduced by Ian Goodfellow et al. in [13] and were quickly adopted for multiple tasks. For instance, in noise-to-image generation, the objective is to sample images starting from a random distribution *z*. In this field, DCGAN [14] presented the first fully convolutional GAN architecture. In addition, BiGAN [15] introduced an inverse mapping for the generated image, and conditional GANs [16] were introduced in order to control the output of a GAN using additional information. Then, BigGAN [17] proved that GANs could successfully generate high-resolution, diverse samples. Recently, StyleGAN [18] and StyleGANv2 [4] generated style codes starting from the noise distribution and used them to guide the generation process, reaching impressive results. Indeed, GANs can be employed to solve several challenges such as text-to-image generation [19], sign image generation [20] or removing masks from faces [21] Another task in which GANs can be used with great success is image-to-image translation, where an image is mapped from a source domain to a target domain. This can be performed both in a paired [22] or an unpaired [23] way. In addition, StarGAN [24] proposed a solution in order to use a single generator even when transferring multiple attributes. However, of these works can be adapted to perform arbitrary style transfer in fashion, as they do not employ style codes as part of the generation process and focus more on performing manipulation over a small set of domains. Finally, with the introduction of Adaptive Instance Normalization [10], several GANs architectures were proposed to perform domain transfer. Some examples are MUNIT [25], FUNIT [26], or StarGANv2 [11]. In particular, StarGANv2 is able to generate images starting from a style extracted from an image or generated with a latent code and represents the main baseline for this work. Nevertheless, these methods are not specifically designed for fashion style transfer, as they also apply changes to the shape of the input image. For this reason, in this paper, we chose to adapt StarGANv2 architecture to perform this new task.
**Neural Style Transfer.** Style transfer has the objective of transferring the style of a target image on a source image, leaving its content untouched. Gatys et al. [27] were the first to use a convolutional neural network to tackle this task. Then, Refs. [28,29] managed to solve the optimization problem proposed by Gatys et al. in real-time. Since then, several other works were proposed [30,31,32,33,34,35]. Then, Chen and Schmid [36] were able to perform arbitrary style transfer, but their proposed method was very slow. None of these methods were tested with fashion images, and they are conceived for transferring styles typically from a painting to a picture and, therefore, cannot be used in this work. On the other hand, for this paper, the fundamental step is represented by the introduction of Adaptive Instance Normalization (AdaIN) [10] that, for the first time, allowed us to perform fast arbitrary style transfer in real-time without being limited to a specific set of styles as in previous works.
**Deep Learning for Fashion.** In the fashion industry, deep learning can be used in several applications. Firstly, the task of the classification of the clothing fashion styles was explored in several works [1,37,38]. Another fundamental topic is clothing retrieval [2,39,40,41], which can be used to find a specific clothes in an online shop using a picture taken in the wild. In addition to that, recommendation systems are also of great interest in order to suggest particular clothes based on the user’s preferences [3,42,43,44,45,46,47]. The task that is most relevant to this paper is that of synthesizing clothes images. This can be achieved by selecting a clothes image and generating an image of a person wearing the same clothes [5,48]. In addition, some works generated clothes starting from segmentation maps [49] or text [6]. Finally, virtual try-on systems have the objective of altering the clothes worn by a single person. Firstly, CAGAN [50] performed automatic swapping of clothing on fashion model photos using cycle-consistency loss. Next, VITON [7] generated a coarse-synthesized image with the target clothing item overlaid on the same person in the same pose. Then, the initial blurry clothing area is enhanced with a refinement network. In addition, Kim et al. [8] performed the try-on disentangling geometry and style of the target and source image, respectively. Furthermore, Jiang et al. ([9,51]) used a spatial mask to restrict different styles to different areas of a garment. Finally, Lewis et al. [52] employed a pose conditioned StyleGAN2 architecture and a layered latent space interpolation method.

Differently from previous methods, our model employs a customized version of StarGANv2 [11]. In addition, the style is not extracted from an art image or a texture, but it is taken directly from target clothes, making the process much more challenging.

## 3. Proposed System

In order to design a model that is able to transfer a clothing style from a source image to a target image, we took inspiration from StarGANv2 [11], but made some modification to its architecture and training. We will describe the architecture and the training in detail in the next sections.

### 3.1. Network Architecture

**Figure 1:**
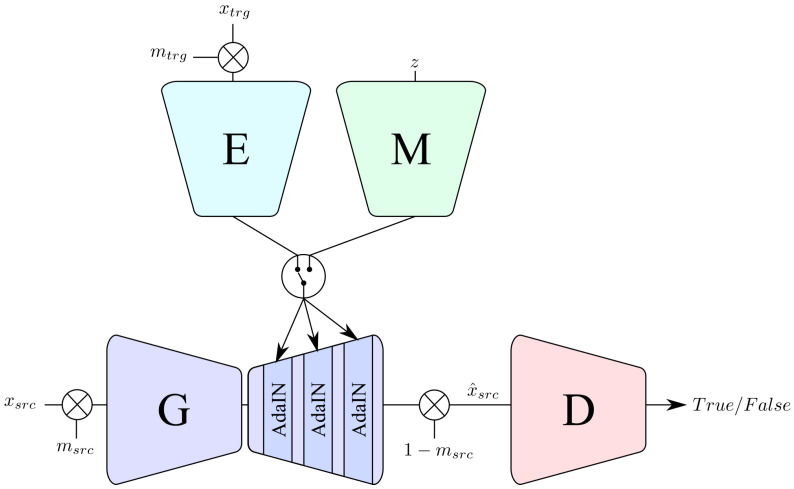
Overview of the proposed system. Firstly, the input image $x_{src}$ is multiplied with its corresponding mask $m_{src}$ and fed to the generator *G*. Then, AdaIN parameters are extracted from either *E* or *M* and injected in the normalization layers of *G*. Finally, the generator output is multiplied again with the inverted mask and used as input to the discriminator *D*.

The proposed model is composed by a Generator *G*, a style encoder *E*, a mapping network *M*, and a Discriminator *D* (see Figure 1). When transferring a style, *E* takes as input a target image $x_{trg}$ and generates a style $s_{trg}$, while *G* takes a source image $x_{src}$ and the style $s_{trg}$ as input generating an output image ${\hat{x}}_{src}$. The style can also be produced from a random noise *z* that is used as input for the mapping network *M* which produces a random style code that can be used by *G* to generate a new image with a custom style. In addition, *D* has the role of evaluating if the generated samples look real or fake, following the well-known GAN paradigm.

More in detail, *G*, as in [11], is an encoder–decoder architecture with four downsampling residual blocks and four upsampling residual blocks. Additionally, the discriminator *D* structure takes inspiration from the StarGANv2 discriminator. However, as several labeled ground-truth domains are not available, we could not take advantage from splitting the discriminator output to classify each domain independently and opted for designing *D* to have a single output. Finally, the style encoder *E* is a single convolutional encoder, while *M* is a multilayer perceptron.

#### 3.1.1. Style Encoder Architecture

**Figure 2:**
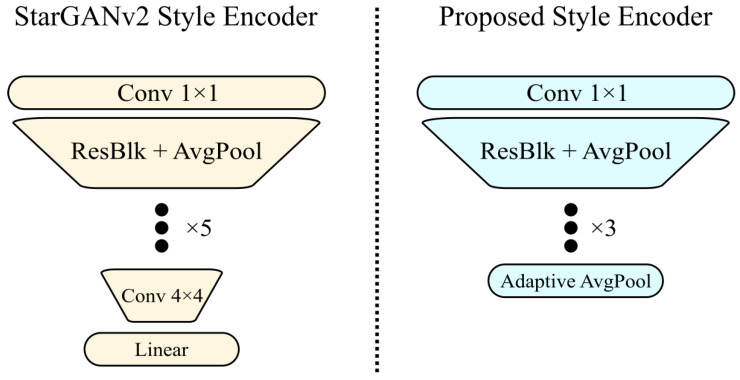
Proposed style encoder architecture. As opposed to StarGANv2, we removed shape information using a pooling function and removing the last two downsampling layers.

Differently from the style encoder of StarGANv2, we heavily modified the architecture of *E* (see Figure 2). In particular, the StarGANv2 encoder did not completely erase the shape information of the reference image, as it needs to change more than just the texture in the source image (such as hair style). On the contrary, we removed this information by inserting a pooling layer and removing two down-sampling layers in the network, as we only need to transfer the clothes style and its shape information is useless.

#### 3.1.2. Transferring a Style with Adaptive Instance Normalization

In order to apply a target style to an image, *G* makes use of AdaIN layers [10]. Specifically, as opposed to standard Instance Normalization (IN) layers, the mean μ and variance σ of the input $x_{src}$ are aligned with ones of the style $s_{trg}$:

(1) $$AdaIN\left(x_{src},s_{trg}\right)=\sigma \left(s_{trg}\right)\left(\frac{x_{src}-\mu \left(x_{src}\right)}{\sigma (x_{src})}\right)+\mu \left(s_{trg}\right)$$

where both $\sigma (s_{trg})$ and $\mu (s_{trg})$ are generated using the style encoder *E* or the mapping network *M*.

Differently from StarGANv2, we only needed to apply the style on a localized area of the source image, which corresponds to the location of the clothes. For this reason, we employed a masking technique in order to achieve this goal. In particular, given masks $m_{trg}$ and $m_{src}$ correspond to the clothes area in the target and source image, respectively, the style transfer equation for our system becomes:

(2) $${\hat{x}}_{src}=G\left(m_{src}\cdot x_{src},E\left(m_{trg}\cdot x_{trg}\right)\right)+\left(1-m_{src}\right)\cdot x_{scr}$$

In more detail, both source and target images are masked in order to apply the style and extract it from only 0the clothes area, and after the translation the background is applied again to the output images. Indeed, clothes masks are provided with the DeepFashion2 [12] dataset, but they can be easily extracted from any image using a human parsing network, as in [53]. Finally, masking the images also has the effect of helping the generalization capabilities of the model, as the network does not have to learn all the possible backgrounds and can tune its parameters only for learning the multiple possible clothes shapes and styles.

### 3.2. Training

In order to train the proposed model, we borrow the training scheme from StarGANv2 and improve it in order to enhance its style transfer capability. First of all, an *adversarial loss* $L_{adv}$ is employed in order to generate realistic results. Differently from StarGAnv2, our discriminator only needs to decide if clothes look real or fake, regardless of its category:

(3) $$L_{adv}=E_{x_{src}} [\log D\left(x_{src}\right)]+E_{x_{trg}} [\log\left(1-D\left({\hat{x}}_{src}\right)\right)]$$

Secondly, a *style reconstruction loss* $L_{sty}$ is introduced in order to prevent the generator *G* from ignoring the style code $s_{trg}$. It is defined as the distance between the style code $s_{trg}$ and the style code extracted from the generated image ${\hat{x}}_{src}$:

(4) $$L_{sty}=E_{x_{src},x_{trg}} [\|s_{trg}-E\left({\hat{x}}_{src}\right)\|]$$

Thirdly, a *style diversification loss* $L_{div}$ serves the purpose of enforcing diversity in the generated outputs (two different style codes should always produce different images):

(5) $$L_{div}=E_{x_{src},x_{{trg}_{1}},x_{{trg}_{2}}} [\|{\hat{x}}_{{src}_{1}}-{\hat{x}}_{{src}_{2}}\|]$$

In this case, the style codes are produced starting from two different random noises, $z_{1}$ and $z_{2}$, fed to the mapping network *M*. Finally, a *cycle consistency loss* $L_{cyc}$ avoids the source image to change its domain-invariant characteristics such as shape and pose:

(6) $$L_{cyc}=E_{x_{src},x_{trg}} [\|x_{src}- [G\left(m_{src}\cdot {\hat{x}}_{src},E\left(m_{src}\cdot x_{src}\right)\right)+\left(1-m_{src}\right)\cdot {\hat{x}}_{src}]\|]$$

In addition to these loss functions, we introduced a new loss term to train the network. In particular, we took advantage of a *perceptual loss* $L_{prc}$ [54] between the target masked image $m_{trg}\cdot x_{trg}$ and the source masked generated samples $m_{scr}\cdot {\hat{x}}_{src}$. The reason is to force the output clothes to be perceptually similar to the target clothes. As a consequence, we managed to perform a better style transfer. Perceptual loss can be written as follows:

(7) $$L_{prc}=\psi \left(m_{scr}\cdot {\hat{x}}_{src},m_{trg}\cdot x_{trg}\right)$$

where ψ is the perceptual distance which is calculated as the difference between a series of weighted activation channels of a pretrained network such as VGG19 [55]. The advantage of a perceptual loss is that is not linked to the pixel difference between the two images, which would be too strong a condition for our purposes. Finally, the full objective function becomes:

(8) $$\underset{G,E}{min}\;\underset{D}{max}\;L_{adv}+\lambda_{sty}L_{sty}-\lambda_{div}L_{div}+\lambda_{cyc}L_{cyc}+\lambda_{prc}L_{prc}$$

where $\lambda_{sty}$, $\lambda_{div}$, $\lambda_{cyc}$, and $\lambda_{prc}$ are the weights of the various losses.

## 4. Results and Discussion

In this section, all experiments performed with the proposed architecture will be presented and discussed. Both qualitative and quantitative results will be evaluated with comments.

### 4.1. Dataset

To train the system architecture, we choose to utilize the DeepFashion2 dataset [12]. It is composed of 491 k images of people wearing 13 categories of clothes: short sleeve top, long sleeve top, short sleeve outwear, long sleeve outwear, vest, sling, shorts, trousers, skirt, short sleeve dress, long sleeve dress, vest dress, and sling dress. The dataset provides additional information for each clothes item such as scale, occlusion, zoom-in, viewpoint, category, style, bounding box, dense landmarks, and per-pixel mask. Images are also divided into commercial and customer types and are taken from several viewpoints and orientations, making it a particularly difficult dataset for a neural network to analyze.

**Figure 3:**
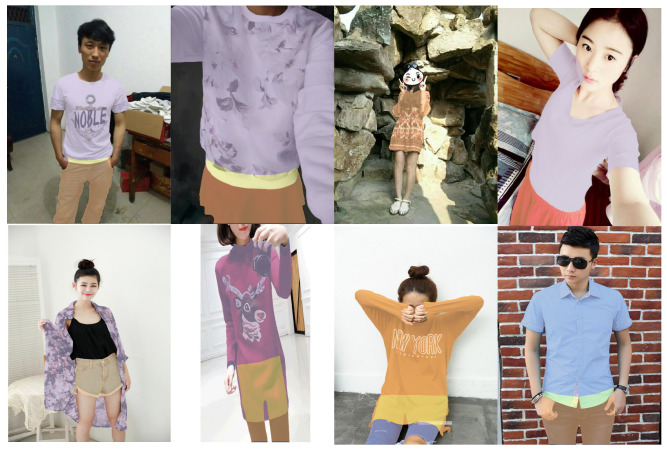
Some images from DeepFashion2 dataset. For each image, a segmentation mask of the clothes is given.

In Figure 3, some images extracted from the dataset are shown. The segmentation mask is the most important feature for our system as it allows us to extract only the style from the target clothes area and apply it only to the source clothes area.

### 4.2. Network Configuration and Parameters

During all the experiments, we trained the model for 50k iterations on an RTX Nvidia Quadro 4000. Training took about 2 days. As an optimizer, we used Adam [56] with a learning rate of 0.0001. During the training, we fixed the parameters as expressed in Table 1. In particular, we chose to employ a style code dimension of 512 instead of 64 (default dimension for StarGANv2) because we empirically found that a higher style dimension is able to express and transfer the style more consistently.

### 4.3. Experiments

**Figure 4:**
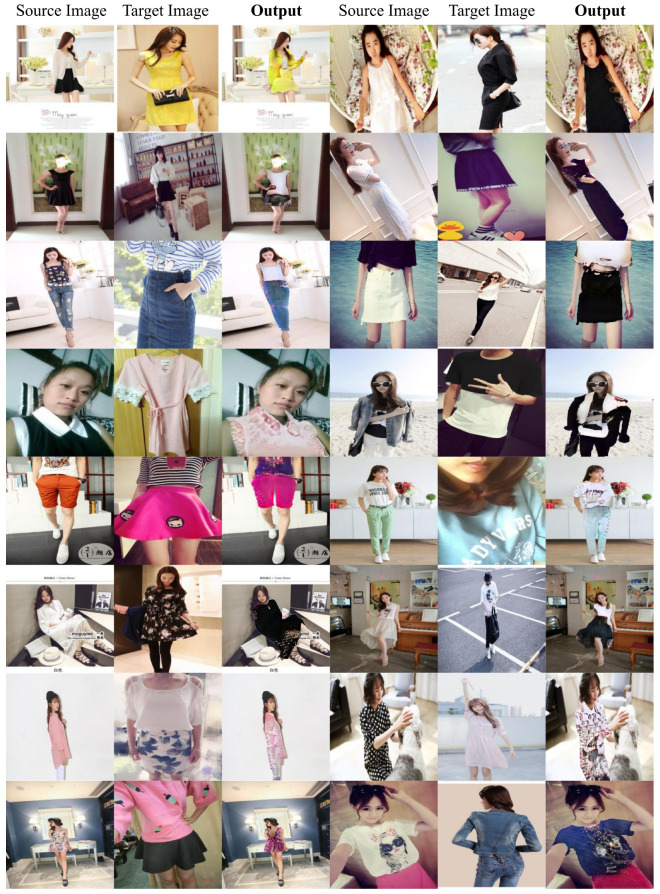
Some results with the proposed method.

We first present some qualitative results in Figure 4. It can be seen how our method is able to apply the style of the target image over the source image even in the very challenging setting of DeepFashion2 dataset. Our system can especially transfer the color style, still being aware of some local information: for example, in the second triplet of the third row, the source image presents a black shirt and a white skirt but, as the target image girl wears a white shirt and black pants, the colors are inverted in the output image. Furthermore, in the first triplet of the third row, the style image contains a light colored shirt and a blue skirt which are then applied in this fashion over the source image. This would not be possible with other older classical computer vision methods such as, for example, histogram matching.

On the contrary, the proposed model still struggles when transferring texture. This is due to the global nature of the AdaIN layers. Indeed, these kinds of normalization layers are not able to apply precise changes over the images and, for this reason, small details in the target images are not transferred over the source images. This is particularly evident for clothes with text or small objects printed on them. Nevertheless, despite not being able to exactly replicate a texture, if the target clothes contain complex texture, the model is still able to generate a result that is not simply a plain color. Indeed, solving this problem can be an interesting future work.

**Figure 5:**
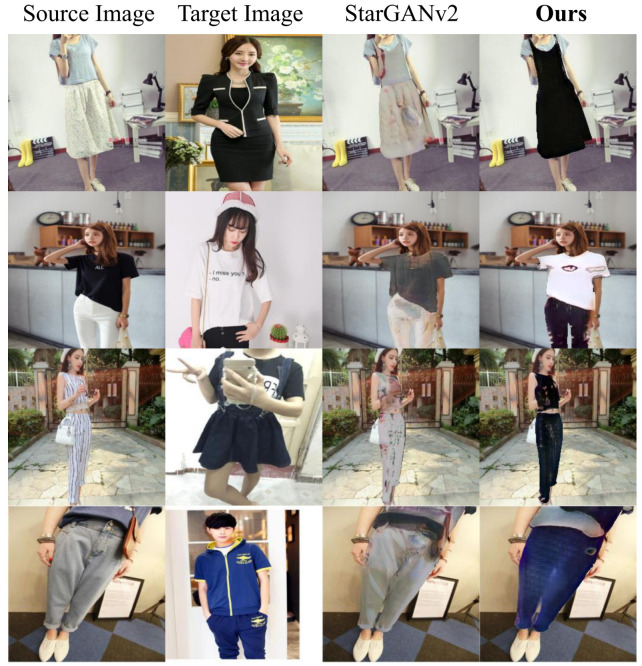
Comparison between baseline StarGANv2 and our method.

After proving the efficacy of our work, in Figure 5 we demonstrate that the baseline StarGANv2 is not suited for the task tackled in this paper. Indeed, StarGANv2 uses a different style encoder network and a smaller style dimension. Looking at the figure, it is clear how it is not able to correctly transfer the clothes style and leaves the output almost identical to the source image. The reason for this is that, given the impressive intrinsic variety of the DeepFashion2 dataset in terms of styles, pose, camera angles, and zoom, the model can not generalize enough in order to produce a meaningful style code that is basically ignored by the generator *G*. On the other side, with the customizations that we introduced, our model overcomes this issue.

Regarding quantitative evaluation, we present the results in Table 2. As in other works on style transfer, we adopted the Learned Perceptual Image Patch Similarity (LPIPS) distance between the source clothes and the generated samples. LPIPS is a metric to evaluate the perceptual similarity of images. It was firstly introduced in [54] for evaluating style transfer methods, and it is based on the idea of comparing intermediate features of deep networks. In [54], several drawbacks of standard measures such as Structural Similarity (SSIM) or Peak Signal to Noise Ratio (PSNR) were identified. It was shown how LPIPS is more reliable and more accurately resembles human perception of similarity between images while being robust to noise. On the contrary, we chose not to use the popular quantitative image quality evaluation metric FID score [57], as our method performs a heavy editing over the images (while StarGANv2 leaves them almost untouched) and this would not be grasped by FID.

**Figure 6:**
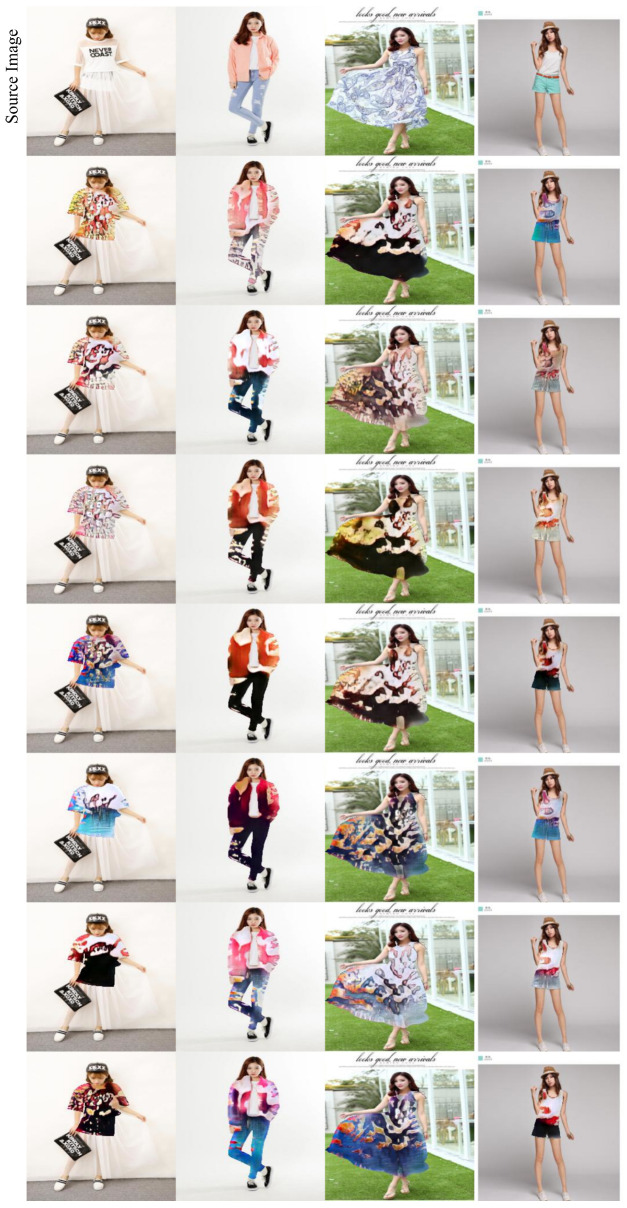
Different styles generated with the mapping network *M*.

Given that our method transfers the style, i.e., manipulates only a region of the image (the clothes), we computed the LPIPS measure for both *(i)* the whole images and *(ii)* the clothes area only. This is intended to highlight the difference in the generated styles. The results in Table 2 indeed show that, with respect to StarGANv2, our method successfully applies a style that makes the generated clothes different from the originals. In addition, in Figure 6 we present some results when the style code is generated starting from a latent code *z* using the mapping network *M*. It is clear how our model is able to generate very different and diverse styles, both in terms of color and motives. Indeed, being able to generate style from random noise is a feature that allows lots of additional freedom in generation and can be of great interest for designers.

**Figure 7:**
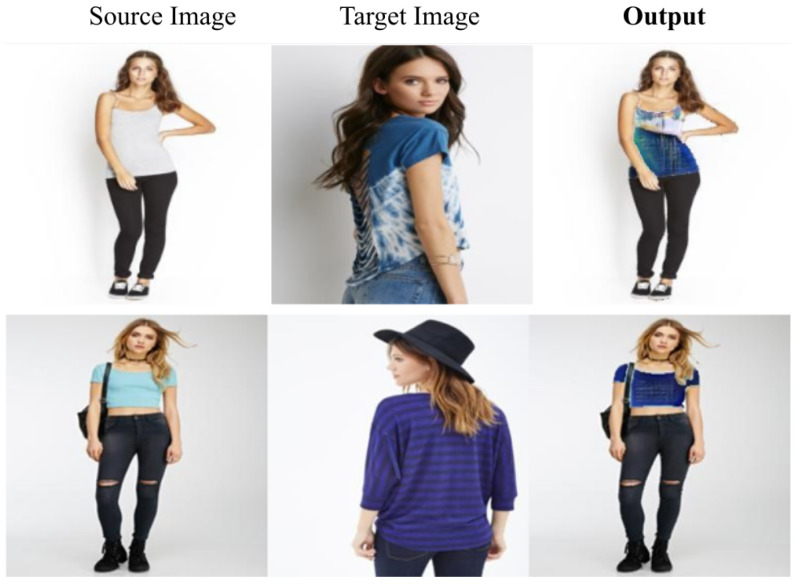
Results with DeepFashion [41] dataset.

In Figure 7, we report some additional qualitative examples using a new dataset. Specifically, we kept the original network weights trained with DeepFashion2 and performed an inference step using images from the original DeepFashion dataset [41]. Indeed, as the DeepFashion dataset does not provide segmentation masks for the clothes area, we generated them using a pretrained human parsing network [53].

### 4.4. Ablation

Finally, we performed several ablations in order to find the best configuration for our method. Table 3 reports the most significant ones. Firstly, we show how with the StarGANv2 style encoder the perceptual distance between source clothes and output decreases. This is due to the better capability of our encoder of transferring the style to the source image. Secondly, we removed the cycle consistency loss to see if $L_{prc}$ would be enough to train the model. Experiments proved that the cycle consistency loss is very important for maintaining the high quality of the style transfer, as it stabilizes the training and maintains the source content and textures during the style transfer. Thirdly, we removed the perceptual loss and, as expected, by doing so the network started struggling when applying the target style, as the generated sample clothes are not bounded to be perceptually similar to the target clothes anymore. Finally, the weight of $L_{prc}$ was reduced, which resulted in worse performance overall.

## 5. Conclusions

In this paper, we presented a novel way to transfer a style extracted from target clothes to a source image containing a person wearing different clothes. This was achieved taking inspiration from the StarGANv2 architecture, but customizing it in order to perform this task. In particular, the style dimension was bigger; the style encoder network was modified, removing two downsampling and adding a pooling layer; and the concept of multiple domains was removed.

Experiments were performed with the challenging DeepFashion2 dataset and proved the efficacy of our method. Nevertheless, results were still not ideal, as the network is not able to correctly transfer texture information and small objects printed on the target clothes due to how AdaIN layers work.

Future works include solving these issues and extending the network to a wider variety of datasets and tasks.

## Figures and Tables

**Table 1:** Parameters employed during training.

Training Parameters	
Style dimension	512
$\lambda_{sty}$	1
$\lambda_{div}$	1
$\lambda_{cyc}$	1
$\lambda_{prc}$	10

**Table 2:** Quantitive comparison between StarGANv2 and our method. Results with and without background are reported separately. (Bold represent the best result.)

	Method	LPIPS ↑
All Image	StarGANv2 [11]	0.524
	**Ours**	**0.551**
Clothes Area	StarGANv2 [11]	0.162
	**Ours**	**0.267**

**Table 3:** Quantitive comparison between results with StarGANv2 and with our method.

	LPIPS ↑
**Ours**	**0.551**
StarGANv2 Encoder	0.540
no $L_{cyc}$	0.542
no $L_{prc}$	0.539
$\lambda_{prc}$ = 1	0.545

## Data Availability

The DeepFashion2 dataset can be downloaded from: https://github.com/switchablenorms/DeepFashion2 (accessed on 20 April 2022).
